# Phytochemical Compositions and Antioxidant Activities of Essential Oils Extracted from the Flowers of *Paeonia delavayi* Using Supercritical Carbon Dioxide Fluid

**DOI:** 10.3390/molecules27093000

**Published:** 2022-05-07

**Authors:** Xiao Yu, Huaibi Zhang, Juan Wang, Junming Wang, Zhenxing Wang, Jinbo Li

**Affiliations:** 1 Faculty of Landscape Architecture and Horticulture Sciences, Southwest Forestry University, Kunming 650224, China; yuxiao19920215@163.com; 2 New Zealand Institute for Plant & Food Research Limited, Private Bag, Palmerston North 11600, New Zealand; huaibi.zhang@plantandfood.co.nz; 3 Eco-Development Academy, Southwest Forestry University, Kunming 650224, China; 4 Faculty of Forestry, Southwest Forestry University, Kunming 650224, China; magina@swfu.edu.cn; 5 Faculty of Life Science, Southwest Forestry University, Kunming 650224, China; wangzhenxingfood@163.com; 6 Dianxiangguose Agricultural Technology Company Limited of Yunnan Province, Kunming 652501, China; 19908718177@163.com

**Keywords:** *Paeonia delavayi*, flower colors, essential oil, antioxidant activity

## Abstract

Essential oils were extracted from dark-purple, red and yellow petals of *Paeonia delavayi* using Supercritical Carbon Dioxide method. The compositions of essential oils were analyzed using gas chromatography-mass spectrometry (GC-MS). Antioxidant activity assays were carried out using DPPH, ABTS- and FRAP methods. Total polyphenols and total flavonoids were measured to evaluate the in vitro antioxidant activity in addition to the volatile compounds contained in the essential oils extracted from the flower petals of *P. delavayi* with the three flower colors. A total of 194 compounds were detected from essential oils of *P. delavayi* flowers, including 83 in dark-purple petals, 90 in red petals and 80 in yellow petals. These compounds mainly include alcohols, aldehydes, ketones, alkenes, alkanes, esters and polyphenols. The results showed that the volatile compounds accumulated differentially among the essential oils from the different colors of flower petals. Principal component analysis (PCA) indicated that essential oils derived from dark-purple and red petals were more closely clustered while the yellow petal essential oil was very different with both the purple-red and red. Antioxidant assays suggested that the radical scavenging activity and the iron reduction antioxidant activity in the essential oils were highly correlated with the flower petal colors. These results suggest *P. delavayi* flower petals are potentially good resources for high quality essential oils and natural antioxidants.

## 1. Introduction

*Paeonia delavayi* belongs to sect. Moutan of Paeonia, which is listed as a rare and endangered perennial woody plant in China [1]. It is mainly distributed in the central and northwestern of Yunnan province, southwestern Sichuan province and southeastern Tibet province [2]. *P. delavayi* plants produce a range of flower colors. Particularly, the gene resource for yellow flower color provides the basis for a very precious flower trait in peony cultivars. Therefore, *P. delavayi* plants have become a very valuable breeding material in the creation of new peony varieties [1]. Meanwhile, peony petals of various colors contain a wide range of polyphenolic compounds and flavonoids [3], including anthocyanins, which provide the power of scavenging superoxide anion free radicals. Peony flowers have been proposed as a potential resource for the development of new drugs or functional foods due to their high healthy and nutritional values [3].

At present, most studies on *P. delavayi* mainly focus on breeding new varieties through hybridization, cultivation, seed production, taxonomy and genetic diversity. These more conventional studies have laid a solid foundation for further research and development into molecular and phytochemistry levels [4,5,6]. 

Traditionally, growers and ornamental industries have economic benefit largely from the flowers, but flowers of many species also produce nectary and fragrances, In *P. delavayi*, flowers also provide most ornamental value, with blooming period being between April and June each year. The flower petals of *P. delavayi* are colorful, showing white, yellow, light red, dark red and dark-purple colors. Yellow, red and dark-purple are the most common colors found in the wild [7,8]. In addition, *P. delavayi* flowers emit refreshing and pleasant fragrances. However, there are few reports on the phytochemical constituents of *P. delavayi* flowers with different flower colors [9,10,11]. 

In this study, essential oils were extracted from *P. delavayi* flower petals of different flower colors, and their phytochemical compositions were detected by gas chromatography-mass spectrometry (GC-MS). The essential oil components and antioxidant activities of different petal colors were compared and analyzed, to provide a fundamental basis for comprehensive utilization and further development of essential oils using this species.

## 2. Results

### 2.1. Physical and Chemical Properties of Essential Oils Extracted from Petals of Different Colored Flowers

The extraction rates, physical and chemical indexes of the essential oils extracted from the petals of the three different flower colors were not meaningfully different (Table S1). The highest extraction rate was 0.78% for dark-purple petals, followed by 0.64% for red petals and 0.58% for yellow petals. Although the essential oils extracted from petals with different colors were cleared, the obtained oil liquid retained the characteristic aroma of the fresh peony flowers. The colors of essential oils from dark-purple and red flower petals manifested milky white while yellow petals was light yellow. The aroma of the essential oil obtained from the yellow petals was more refreshing and elegant than that of dark-purple and red petals. There was no significant difference in the relative density, refractive index and optical rotation of essential oils among the essential oils derived from the three colors.

### 2.2. Comparison of Volatile Profiles among Essential Oils from Different Colors by Total Ion Chromatograms

The total ion chromatograms of volatile components in *P. delavayi* essential oils from different flower colors were examined with GC-MS analysis using 1.0 μL injection volume of essential oils (Figure S1, Supplementary Materials). While the fractionation time of essential oils from dark-purple, red and yellow petals was concentrated in 15–60 min, the predominant peaks of components in dark-purple and red petals were more concentrated between 15–30 min, and the peaks accounted for 38.46% and 30.19% of the total peaks, respectively. The fractionation peaks from yellow petals were relatively more dispersed, accounting for only 28.23%.

Significant differences of chemical components and their relative contents in the three essential oils were revealed by the GC-MS (Table S2). A total of 194 compounds were obtained from the essential oil extractions of three colors petals of *P. delavayi*. Among them, 83 were found in dark-purple petals, 90 in red petals, and 80 in yellow petals. The types of compounds with relatively large contents in the essential oil extracts of the three petals are different. Among them, the main compositions with relatively large content in the essential oil of dark-purple petal were identified as 2-(5-Ethenyl-5-methyloxolan-2-yl)propan-2-ol (20.88%), 2H-Pyran-3-ol, 6-ethenyltetrahydro-2,2,6-trimethyl-, (3R,6S)-rel- (11.90%), benzenepropanal(8.46%), cinnamaldehyde, (E)- (4.92%), and phytol (4.34%). In the red petals, essential oil contained 2-(5-Ethenyl-5-methyloxolan-2-yl) propan-2-ol(21.81%), (E)-linalool oxide (pyranoid) (10.85%), β-methylenephenethyl alcohol(5.60%), cyclooctyl isopropylphosphonofluoridate(5.16%), and trans-cinnamaldehyde(4.31%). In the yellow petals, essential oil had tricycle [4.4.0.02,7]decane, 1-methyl-3-methylene-8-(1-methylethyl)-, (1R,2S,6S,7S,8S)-rel-(20.34%), 2-naphthalenemethanol, decahydro-8-hydroxy-α,α,4a,8-tetramethyl-(7.9%), dl-1-Phenethylalcohol (5.97%), 2-(4-methylidenecyclohexyl)prop-2-en-1-ol (5.35%), and N-Hexadecane (4.36%).

### 2.3. Comparative Analysis of Volatile Compounds in Essential Oil of P. delavayi with Different Flower Colors

#### 2.3.1. Analysis of Common VOLATILE Compounds in Essential Oils of *P. delavayi* Petals with Three Colors

The essential oils extracted from the flower petals with three different colors of *P. delavayi* had 11 compositions detected in common (Table 1). The percentage of common compositions in total accounted for 50.46% for dark-purple petals, 49.47% for red petals and 22.9% for yellow petals. In addition to the 11 common compounds, the dark-purple petals and the red petals also shared 18 compounds, namely 3-phenyl-1-propanol, (βR,2S,5S)-β,5-dimethyl-5β-vinyltetrahydrofuran-2α-ethanol, epoxy-linalooloxide, cinnamyl alcohol, 2,6-dimethyloct-7-ene-2,6-diol, 4,4-dimethyladamantan-2-ol, 3-methylbut-2-enoic acid, 3-fluorophenyl ester, palmitic acid ethyl ester, 1-bromo-1,2-dichlorocyclopropane, 2,6,10-trimethyldodecane, 4-methoxy-3-hydroxyacetophenone, 1-nonanal, 6,6-dimethylbicyclo[3.1.1]heptane-2-carbaldehyde, phenylpropyl aldehyde, (1E,6E,8S)-1-methyl-5-methylene-8-isopropyl-1,6-cyclodecadiene, 2,7-octadiene-1,6-diol,2,6-dimethyl-, bicyclo[5.2.0]nonane, 4-ethenyl-4,8,8-trimethyl-2-methylene-, and 2(5H)-Furanone,5-(1-methylethyl)-. Dark-purple petals and yellow petals also shared another nine common compounds, such as trans-3-hexen-1-ol, 3-isopropyl-6,7-dimethyltricyclo[4.4.0.0(2,8)]decane-9,10-diol, 1-cyclopropylpropane, 8,8-diheptylpentadecane, 4-chloro-4′-hydroxybutyrophenone, 2-methylpent-4-enal, trans-2-hexenal, 2,7-octadiene-1,6-diol, 2,6-dimethyl-, (2Z)-, and 1-benzothiophene-3-carboxylic acid. Red and yellow petals shared 11 compounds, benzyl alcohol, dl-1-phenethylalcohol, linalool, phenethyl alcohol, acetic acid, 2,2,2-trifluoro-, 5-methyl-2-(1-methylethenyl)-4-hexen-1-yl ester, pentanoic acid,5-hydroxy-,2,4-bis(1,1-dimethylethyl)phenyl ester, 3,3-dimethylhexane, n-hexadecane, carissone, cis-muurola-4(14),5-diene, 2-naphthalenemethanol, and decahydro-8-hydroxy-α,α,4a,8-tetramethyl. The analysis of these common compounds not only shows the composition differences between different sources of *P. delavayi* essential oils with different colors, but also can be used as a data support for *P. delavayi* classification.

#### 2.3.2. Differences in Chemical Classes of the Essential Oils from the Three Colors of Flower Petals in *P. delavayi*

**Figure 1:**
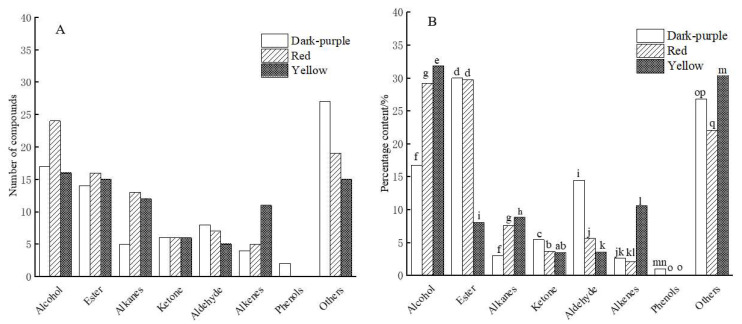
Comparison of number and content of different types of compounds in *P. delavayi* essential oil with different flower colors. (**A**): Number of compounds. (**B**): Content percentage of compound. Different lowercase letters in the same class of compounds in the figure indicate significant differences (*p* < 0.05).

According to chemical classification, the compounds detected in the essential oils of the three different colors belonged to 8 types of compounds, namely alcohols, esters, alkanes, ketones, aldehydes, alkenes, phenols and others. Figure 1A,B, respectively, compare and analyze different classes of compounds.

According to the chemical properties and analysis of the identified chemical constituents, 17 alcohols (16.72%), 14 esters (29.99%), 8 aldehydes (14.42%), 6 ketones (5.45%), 5 alkanes (2.99%), 4 alkenes (2.64%) and 2 phenols (1%) were identified in the essential oil of dark-purple *P. delavayi* flowers. Red *P. delavayi* petal essential oil had 24 alcohols (29.18%), 16 esters (29.76%), 13 alkanes (7.59%), 5 alkenes (2.12%), 6 ketones (3.64%), 7 aldehydes (5.60%), 16 alcohols (31.88%), and 15 esters (7.99%). In the yellow color, 12 alkanes (8.85%), 11 alkenes (10.58%), 6 ketones (3.44%) and 5 aldehydes (3.55%) were detected in the essential oil. The special fragrance of peony flower may be related to its abundant alcohols, esters and alkanes.

The relative percentage of alcohol substances in the essential oil of yellow *P. delavayi* is the highest. The alcohol content of the essential oil in the yellow and red petals displayed about 2% difference, which was significant (*p* < 0.05). The essential oil derived from the dark-purple *P. delavayi* had much lower alcohol than that in the red or yellow (*p* < 0.05) (Figure 1B). Alcohols usually provide soft aromatic and plant aroma. The percentage content of alkanes in essential oils from yellow petals was also significantly higher than that from red petals and dark-purple petals (*p* < 0.05). In terms of aldehydes, the essential oils of dark-purple petals were significantly higher than those of red petals and yellow petals (*p* < 0.05). In terms of esters, the percentages of *P. delavayi* dark-purple flowers and red flowers were similar (*p* > 0.05) and significantly higher (*p* < 0.05) than those of yellow flower essential oils. The content of ketones in dark-purple *P. delavayi* petal essential oil was the highest, while the content of ketones in red and yellow *P. delavayi* petal essential oil was similar, with non-significant differences (*p* > 0.05). Additionally, there was no significant difference in the contents of alkenes among the three *P. delavayi* essential oils (*p* > 0.05). Phenolic compounds only exist in the essential oil of *P. delavayi* dark-purple petals.

#### 2.3.3. Principal Component Analysis

Principal component analysis (PCA) uses a mathematical dimensionality reduction method to replace many original variables with several comprehensive variables. Therefore, these comprehensive variables can represent the information of all the original variables without causing collinearity problems [12]. The principal component analysis of 11 common compounds was carried out by SPSS17.0 to explore the main components of *P. delavayi* essential oils with different colors. The eigenvalues and contribution rates of 11 common compounds are shown in Table S3 and the results of component load matrix analysis are shown in Table S4. According to the principle of eigenvalue greater than one, two principal components were analyzed, and the contribution rates were 67.528% and 32.472%, respectively. The cumulative contribution rate reached 100%, indicating that the two principal components can reflect most of the information of the original variable. The analysis results reduced the 11 common compounds in the original *P. delavayi* flowers to two irrelevant principal components, achieving the purpose of dimension reduction.

According to the analysis in Table S4, the positive influence compounds with higher load in the first principal component (PC1) were mainly benzaldehyde, trans-Cinnamaldehyde, 2-(5-Ethenyl-5-methyloxolan-2-yl)propan-2-ol, (E)-linalool oxide (pyranoid), and the negative influence compounds with higher load were phenethyl alcohol, α-cadinol, (S,3E,7E)-α,α,4,8-Tetramethyl-3,7-cyclodecadiene-1-methanol. The positive influence compounds with higher load in the second principal component (PC2) were mainly benzyl alcohol, phytol, bicyclo[3.2.2]nona-6,8-dien-3-one, and the negative influence compounds with higher load were phenethyl alcohol and fitone.

**Figure 2:**
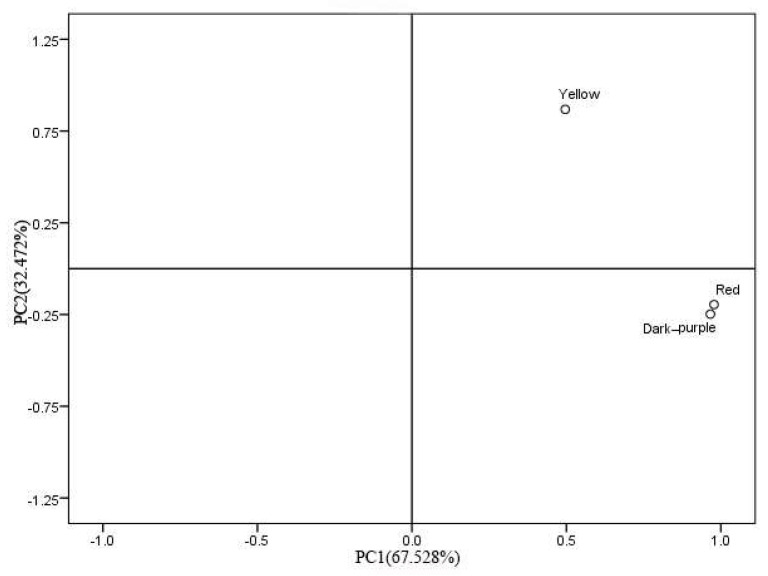
Sample score chart.

**Figure 3:**
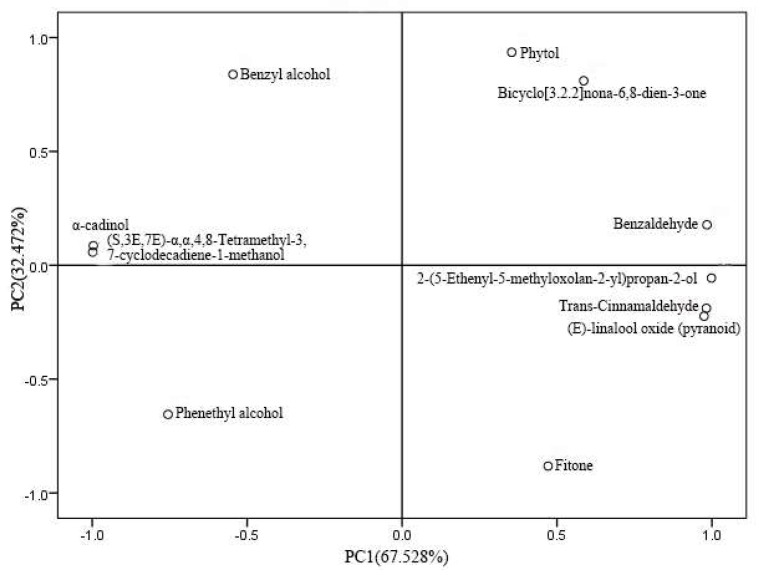
Load diagram of 11 components on the principal component.

The principal component analysis of three kinds of flower essential oils was carried out by SPSS17.0 software. According to the principle of eliminating the variable of the maximum eigenvector corresponding to the principal component of the minimum eigenvalue, one variable was eliminated each time, and then the principal component analysis was carried out on the remaining variables. The sample score diagram (Figure 2) and the principal component load diagram (Figure 3) were obtained, respectively. Analysis of the essential oil scores of different flower colors (Figure 2) found that the highest score of PC1 was red flowers, and the highest score of PC2 was yellow flowers. The *P. delavayi* essential oils with dark-purple and red petals are distributed in the same quadrant, while the yellow petals are distributed in other quadrants, indicating that the essential oils of yellow petals are quite different from those of dark-purple and red petals. It can be seen from the principal component load diagram (Figure 3) that 11 compounds are scattered in each quadrant. According to the analysis of sample score diagram and principal component load diagram, (E)-linalool oxide (pyranoid), trans-cinnamaldehyde and 2-(5-Ethenyl-5-methyloxolan-2-yl)propan-2-ol may be the main components of dark-purple and red petal essential oils. Phytol, while benzaldehyde and bicyclo[3.2.2]nona-6,8-dien-3-one may be the main components of yellow petal essential oil [13,14,15].

### 2.4. Antioxidant Properties

#### 2.4.1. DPPH Radical Scavenging Activity

**Figure 4:**
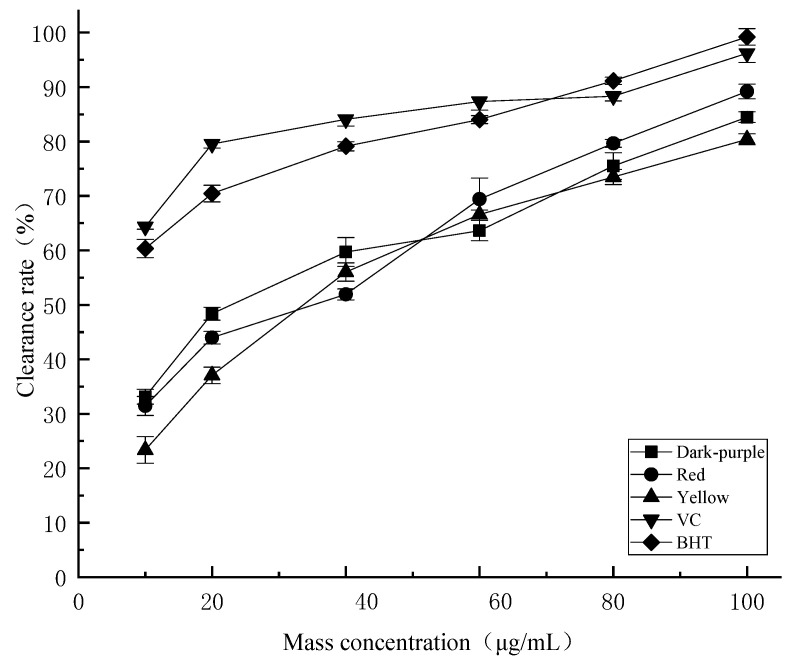
Comparison of the DPPH radical scavenging activities of the essential oil for different colors of *P. delavayi* flowers.

DPPH is a stable nitrogen-centered proton free radical in organic solvents, and its scavenging capacity is significantly positively correlated with its antioxidant capacity. From Figure 4, it can be seen that essential oils from *P. delavayi* flowers with different colors have obvious scavenging effects on DPPH free radicals. In the mass concentration range of 10–100 μg/mL, the DPPH free radical scavenging activity increases with the increase of concentration. In the test concentration range, the DPPH scavenging activities of essential oils from different flower colors were lower than those of the positive control ascorbic acid (V_C_) and butylated hydroxytoluene (BHT). When the concentration of essential oil reached 100 μg/mL, the DPPH free radical scavenging activity of essential oils with different flower colors could reach 70% of that of the positive control at the same concentration. The scavenging activity of essential oil from red petals (89.19% ± 1.35) was significantly higher than that from dark-purple petals (84.47% ± 0.98) and yellow petals (80.4% ± 1.01). The IC_50_ values of essential oils from dark-purple, red and yellow *P. delavayi* flowers for DPPH radical scavenging activity were 30.49 μg/mL, 33.61 μg/mL and 41.07 μg/mL, respectively.

#### 2.4.2. ABTS Radical Scavenging Activity

**Figure 5:**
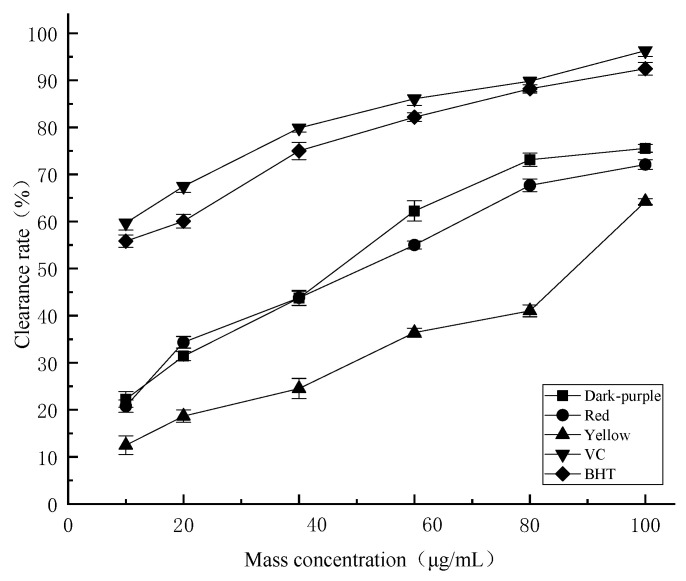
Comparison of the ABTS radical scavenging activity of essential oil for different colors of *P. delavayi* flowers.

ABTS radical scavenging activity is a widely used method for determining the total antioxidant capacity of biological samples. It is easy to operate and rapid. It can be seen from the results (Figure 5) that *P. delavayi* essential oils with different colors both have better ability to scavenge ABTS, and the scavenging effect is lower than that of the positive controls ascorbic acid (V_C_) and BHT. ABTS scavenging activity increased with the increase of essential oil concentration. The scavenging activity increased slowly before the concentration of 40 μg/mL, indicating that there was not only a dose-dependent relationship between the sample volume and the scavenging effect, but also a significant dose-dependent effect. When the essential oil concentration reached 100 μg/mL, the scavenging activity of dark-purple petal essential oil (75.55% ± 0.84) was significantly higher than that of red (72.11% ± 1.01) and yellow (64.25% ± 0.58) petals. The IC_50_ values of ABTS scavenging capacity of essential oils from dark-purple, red and yellow flowers of *P. delavayi* were 49.231 μg/mL, 53.565 μg/mL and 84.010 μg/mL, respectively.

#### 2.4.3. Ferric Reducing Antioxidant Power

**Figure 6:**
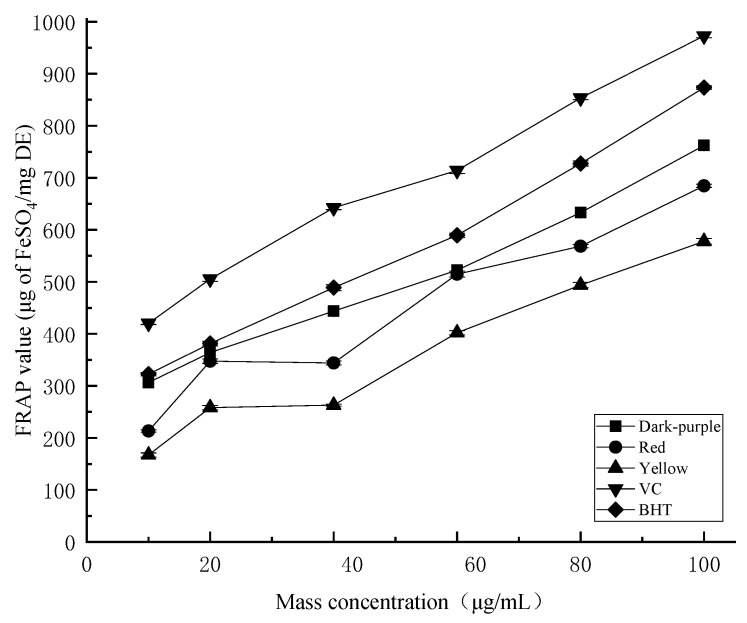
Ferric reducing antioxidant power of essential oil for different colors of *P. delavayi* flowers.

Through the FeSO_4_ standard solution, the linear equation of the total reducing ability standard curve was y = 0.0114x + 0.022, R^2^ = 0.9994. The ferric reducing antioxidant activity of the tested samples increased with the increase of volume (Figure 6). When the substrate concentration changed from 10 to 100 μg, the FRAP value showed a good linear relationship with the volume of the sample. This indicated that essential oils from different flower colors of *P. delavayi* had high ferric reduction antioxidant activity, but the antioxidant activity was lower than that of the positive control ascorbic acid (V_C_) and BHT. When the concentration of essential oil reached 100 μg/mL, the ferric reducing antioxidant activity of dark purple petal essential oil (762.03 ± 4.84 μg/mg) was significantly higher than that of red (684.53 ± 3.01 μg/mg) and yellow (577.94 ± 5.58 μg/mg) petals.

### 2.5. Total Phenolic and Flavonoid Content

Use the absorbance value to make a standard curve for the concentration of gallic acid standard solution: y = 0.0687x + 0.0443, R^2^ = 0.9946. The standard curve was made by absorbance value to the concentration of rutin standard solution: y = 0.9489x − 0.0053, R^2^ = 0.9996. As shown in Table 2, the contents of total phenols and flavonoids in dark purple petals were the highest, which were 28.71 ± 0.42 mg/g and 10.8 ± 0.06 mg/g, respectively, followed by red petals and yellow petals.

## 3. Discussion

### 3.1. Chemical Compositions in the Essential Oils Are Differentially Accumulated in Relation to the Flower Colors in P. delavayi

Peony is a traditional and well-known ornamental plant in China. It is widely used as a garden and medicinal plant as well as providing ecological values, and it is suitable for developing functional foods [16]. The wide distribution of *P. delavayi* in Southwestern China led to the diversity in phenotypic traits such as leaf lobe number, leaf lobe width, flower colors, carpel and bract number [17]. Some studies have analyzed the metabolome of flavonoids of *P. delavayi*, and differential accumulation of the metabolites between dark-purple flowers, red flowers and yellow flowers was reported [3]. While the difference between dark-purple flowers and red flowers is small, the differences between the yellow color and other colors are large. This reflects similarity of the metabolic pathways between the red and dark-purple flowers and a divergence of the yellow flowers. These findings are consistent with the results of this study.

According to the composition analysis on the essential oils by GC-MS and SPSS, *P. lutea* and *P. rockii* contain more terpenoids than other essential oil compositions, mainly oxidized linalool, isophytic alcohol, and farnesol and phytic alcohol [18]. Additionally, the compositions of *P. suffruticosa* essential oil extracted by hydro distillation extraction, ultrasonic assisted hydro distillation extraction and ultrahigh pressure extraction were compared [19]. The common compositions in the essential oils extracted by the three methods contained a large number of aroma compounds, such as linalool and its oxides, cumin alcohol, nerol, geraniol, lauryl alcohol, etc. [19]. These results are in line with our results in this study, i.e., aroma alcohols have the largest representation in the essential oils extracted from the three flower colors of *P. delavayi* (Figure 1).

The percentage content of alkanes in *P. delavayi* essential oils from yellow petals was significantly higher than that from red petals and dark-purple petals (*p* < 0.05). This is represented by β-Copaene (20.34%), a sesquiterpene hydrocarbon. Although alkane compounds generally have a higher threshold value and have little contribution to the overall odor [20], β-Copaene presents in many essential oils and could become an influential factor of essential oils and other applications [21,22].

The higher percentage content of hydrocarbons may be due to the protective wax layer on the petal surface, and the main component of the wax layer was alkane compounds [23]. In terms of aldehydes, the essential oils of dark-purple petals showed significantly higher content than those in the red petals and yellow petals (*p* < 0.05). Aldehydes can provide stronger volatile and fat aroma [20].

Many intrinsic factors affect the color of flowers, such as regulations of metabolic pathways for pigments (e.g., anthocyanins, carotenoids, flavonoids), influenced also by ecological factors. These factors comprehensively influence the biosynthesis, accumulation and stability of anthocyanins that lead to different colors [24,25]. These factors also affect the compositions of essential oil extracted from petals, resulting in a differential accumulation in different colors of flowers in *P. delavayi*.

Among the detected common ingredients, benzyl alcohol, phenethyl alcohol, α-cadinol, benzaldehyde and trans-cinnamaldehyde are all good spice substances. Phenethyl alcohol has which has sweet rose-like fragrance, and it is an edible spice permitted in USA. It has been widely used in the production of food processing industry [26]. Trans-cinnamaldehyde, as a hydroxy acid fragrance-containing compound, has a good fragrance holding effect. It is used as a raw material for flavoring to make the aroma of the main spices clearer. At the same time, it can be sterilized, disinfected and antiseptic in medical applications, especially for fungi. It has anti-ulcer effect, strengthening stomach and intestinal movement effect. It has been widely used in floral flavors for daily use [27]. Therefore, *P. delavayi* essential oils with different colors have a great applicable prospect in food, fragrance, medicine and other industries. These three kinds of essential oils extracted from flowers with different colors contain a variety of valuable chemical components, and can serve as the basis for further development and utilization. Particularly the high level of copaene in the yellow flower holds a promising potential in the future research and development.

### 3.2. Essential Oils of P. delavayi Possess Significant Antioxidant Activities Influenced by Polyphenolic and Flavonoids

Free radicals can lead to the aging of human cells and many diseases, such as cancer and heart disease [28,29,30,31,32]. Since phenols and flavonoids extracted from plants can effectively mitigate free radicals in the body, they can help prevent and treat related diseases caused by free radicals [33,34,35,36].

A large number of literature review shows that flowers contain certain antioxidant activity, and there are significant differences in antioxidant activity among different flowers. The antioxidant activity of peony is relatively strong [37], which is related to its rich active substances. In this study, three antioxidant capacity methods (DPPH, ABTS and FRAP) were used to analyze and measure the extracts of purple, red and yellow flower petals of *P. delavayi*. Our results have shown the essential oils from all three colors of flowers provide good level of antioxidant activity, with the dark-purple flowers displaying the strongest. A previous study had also shown that purple flowers produced highest antioxidant activity [38], in agreement with our result. However, their measurements showed a higher antioxidant activity in the yellow than the red flowers they used [38]. The antioxidant activities of essential oils of the 14 peony samples measured by DPPH and ABTS assays showed that *P. lutea* had the strongest activity in the DPPH and ABTS, 306.42 TE µmol/100 g (DW) and 755.83 TE µmol/100 g (DW), respectively [18]. Previous studies revealed that scavenging activities of DPPH and ABTS free radicals from essential oil of *P. lactiflora* flower increased from morning-picked to afternoon-picked flowers [39]. Compared with this study, the scavenging effect of DPPH of *P. delavayi* is higher than that of V_C_ and *P. lactiflora*,, but the scavenging effect of ABTS was weaker than that of V_C_ and *P. lactiflora.*

The study on the total phenol content of 23 edible flowers showed that the total polyphenols content of 22 flowers ranged from 4.83 to 68.19 mg·g^−1^ (FW) [40], while that of *P. lactiflora* was 222.01 mg·g^−1^ (FW), showing that the total phenol content in the petals of *P. lactiflora* is higher than that of other edible flowers. The contents of total flavonoids in the petals of different varieties of *P. lactiflora* were 4.90–11.00 mg·g^−1^ (FW) [41]. In this study, the content of total flavonoids in different flower colors of *P. delavayi* was 5.45–10.8 mg of RE (rutin equivalent)/g. Compared with different flower colors of *P. delavayi*, the content of total polyphenols was lower, while the content of total flavonoids was close to that in *P. lactiflora*. Total flavonoids were also expressed at a high level in some plant essential oils. The total flavonoid content of essential oils using water distillation extracted from *Citrus reticulata* peel [42], untan pumelo (*Citrus grandis* Osbeck) peel [43], aerial parts of *Teucrium alyssifolium* [44], and *Laurocerasus phaeosticta* [45] leaves were 14.63 ± 0.95 mg CE (catechin equivalents)/g, 134 mg QE(quercetin equivalents)/g, 16.82 mg of Ru(rutin equivalent)/g, and 37.53 mg of RE(rutin equivalent)/g, respectively. The total flavonoid content of *Lycium europaeum* fruit essential oil extracted with supercritical carbon dioxide can also reach 6.8 mg QE(quercetin equivalents)/g [46]. Studies have shown that *P. delavayi* is rich in flavonoids, including isolirin, kaempferol, quercetin, isorhamnetin, land eugenol and apigenin glucoside [3]. Through the transcriptome sequencing analysis of yellow and purple black flowers of *P. delavayi*, the color of yellow and purple red petals of *P. delavayi* is closely related to flavonoids. Using KEGG database, many single genes correspond to the pathways involved in the biosynthesis of secondary metabolites and anthocyanin deposition, including ‘flavonoid biosynthesis’ (179,0.74%), ‘flavonoids and flavonol biosynthesis’ (100,0.41%), ‘anthocyanin biosynthesis’ (10,0.04%) and ‘isoflavone Biosynthesis’ (65,0.27%). Overall, 3969 of 18,784 DEGs were mapped to 127 pathways. It was pointed out that 59, 39 and 5 DEGs were involved in ‘flavonoid biosynthesis’ (ko00941), ‘flavonoid and flavonol biosynthesis’ (ko00944) and ‘anthocyanin biosynthesis’ (ko00942), respectively, and all belonged to the flavonoid biosynthesis pathway. The research shows that the accumulation of flavonoids plays an important role in the formation and development of flower color of *P. delavayi* [1].

Additionally, different colors have different biochemical components. Studies have shown that flavonoid and polyphenolic accumulation in yellow and purple *Passiflora caerulea* fruit are significantly different [47]. The antioxidant capacity of *P. delavayi* petals with different flower colors, the total polyphenolic content and total flavonoids content can be used as a selection criterion for functional food [48]. In this study, we have clearly shown the positive correlation between the polyphenolic, flavonoids content in the essential oils of three different colored flowers (Table 2) and their antioxidant capacities (Figure 4, Figure 5 and Figure 6). These results provide an insight into future applications of peony essential oils in food, medicine and/or skin care fields.

## 4. Materials and Methods

### 4.1. Materials and Reagents

The dark-purple, red, and yellow fresh petals of *P. delavayi* (Figure S2) were collected from the collection garden of *P. delavayi* germplasm resources located in Liangwang mountain, Chengjiang county, Yuxi city, Yunnan province, China (coordinates:102°53′55″ E, 24°45′38″ N, attitude: 2733 m). The samples were refrigerated at 4 °C and shipped to the laboratory for sampling and analysis.

All chemical reagents including anhydrous ethanol, potassium persulfate (K_2_S_2_O_8_), ferric chloride (FeCl_3_), acetic acid (CH_3_COOH), sodium acetate (CH_3_COONa), sodium nitrite (NaNO_2_), sodium hydroxide (NaOH), aluminum nitrate (Al(NO_3_)_3_) were purchased from Sinopharm Chemical Reagent Co., Ltd. (Shanghai, China). 1,1-diphenyl-2-trinitrophenylhydrazine (DPPH), 2,2′-diazo-bis-3-ethylbenzothiazoline -6-sulfonic acid (ABTS), 2,4,6-tripyridyltriazine (TPTZ), and other analytical grade chemicals were purchased from Aladdin (Shanghai, China). Rutinum and gallic acid standards were purchased from Yuanye Bio-technology (Shanghai, China).

### 4.2. Main Instruments and Equipment

SFE2-supercritical carbon dioxide fluid extractor (Applied Separations (ASI), Los Angeles, CA, USA); GC-MS (gas chromatograph, Agilent company, San Francisco, CA, USA; 7890A mass spectrometer, Waters company, Milford, CT, USA);N-1200B Rotary Evaporator (Shanghai Quanjie Instrument Co., Ltd., Shanghai, China).

### 4.3. Essential Oil Extraction

First, 120 g of fresh petals of different colors were placed ut into the extraction tank. The carbon dioxide is pressurized to the extraction tank by a pressurizing pump. The extraction temperature is 40 °C, the extraction pressure is 25 MPa, the carbon dioxide flow rate is 15 L/h, and the entrainer is anhydrous ethanol. When the pressure reached the set pressure, the cycle extraction was started. The extraction time was set as 3 h, and the extracted essential oil was collected every 1 h for three times [49,50,51]. After the extraction, the translucent oily extracts were combined and mixed and collected, then concentrated by rotary evaporation with a rotary evaporator, after which they were weighed and the extraction yield was calculated. Extraction rate (%) = quality of essential oil/quality of petals (dry weight) × 100%.

### 4.4. GC-MS Composition Detection

Gas Chromatography (GC) analysis: The essential oil was dissolved in anhydrous ethanol to prepare a solution with a concentration of 5.0 mg/mL for GC/MS testing. The essential oil was analyzed by using an Agilent Technologies HP 6890 Plus Gas chromatograph. The analysis was carried out on an HP-5MS column (Agilent Technologies, Santa Clara, CA, USA) measuring 30 m × 0.25 mm, film thickness 0.25μm. The carrier gas as hydrogen was at a flow rate of 1 mL/min. The injector temperatures were maintained at 250 °C. The column temperature was programmed from 40 °C with a 2 min hold to 220 °C with 10 min at 4 °C/min. A volume of 1.0 mL of each oil sample was injected into the GC by splitting method with the split ratio of 10:1. The inlet pressure was maintained at 6.1 kPa. The relative amounts of each component were calculated based on the GC peak area percentage that was using the GC HP-Chemstation software (Agilent Technologies, Santa Clara, CA, USA).

Gas Chromatography-Mass Spectrometry (GC-MS) analysis: The electron energy of electron bombardment (EI) ion source is70 eV. The voltage of the electronic multiplier is 1.00 kV. The scanning range of mass charge ratio is 30~550. The temperature of ion source and interface is 250 °C.

Identification of components: The acquired mass spectra were searched using NIST11 and NIST11s standard mass spectral libraries, and combined with the retention index for comprehensive characterization. The percentage content of each compound was calculated by the normalization method of chromatographic peak area.

### 4.5. Determination and Analysis of Physical and Chemical Indicators of Essential Oils

Refer to ISO212:1973 and ISO279:1998 for the nomenclature principle of essential oils and the determination of relative density; refer to ISO280:1998 and ISO592:1998 for the determination of the refractive index and optical rotation of essential oils.

### 4.6. Determination of Antioxidant Capacity of Different Flower Colors

#### 4.6.1. Determination of the Ability to DPPH Free Radical Scavenging

At the condition of room temperature, 100 μL of the sample that was diluted to the appropriate concentration was fully mixed with 100 uL of DPPH (1, l-diphenyl-2-picrylhydrazyl) solution in a 96-well microplate reader. After dark reaction for 30 min, the absorbance (*A*) was measured at 517 nm. The reaction with 70% ethanol solution of equal volume instead of the sample was taken as blank (*A*_0_). The reaction of equal volume of 70% ethanol solution instead of DPPH solution was used as the sample blank (*A*_1_), and 10 μg/mL, 20 μg/mL, 40 μg/mL, 60 μg/mL, 80 μg/mL and 100 μg/mL ascorbic acid (Vc) and quercetin (BHT) were used as the positive control. The inhibition percentage of DPPH free radicals was calculated as inhibition percentage(%) = [*A*_0_ − (*A* − *A*_1_)]/*A*_0_ ∗ 100. Origin 2018 was used to perform nonlinear curve fitting between the clearance rate and the sample concentration (μg/mL) to calculate the IC_50_ value (the sample concentration when the DPPH free radical clearance rate was 50%). The DPPH free radical scavenging ability of the samples and the positive control was expressed by the IC_50_ value [52].

#### 4.6.2. Determination of the Ability to ABTS Free Radical Scavenging

At room temperature, add 50 μL of sample and 200 μL of ABTS (7 mM) solution to a 96-well microtiter plate and mix well, and react in the dark for 6 min. After that, the absorbance value (*A*) was measured at 734 nm, the reaction of replacing the sample with 70% ethanol solution was the blank (*A*_0_), and the reaction of replacing the ABTS solution with 70% methanol solution was the sample blank (*A*_1_), which was repeated 3 times. The generally used formula of DPPH assay was used for the calculation of the percentage of inhibition of ABTS radicals. Free radical scavenging activity is expressed as IC_50_ value, the essential oil concentration (μg/mL) at which 50% of ABTS free radicals are inhibited. The IC_50_ value was calculated from the linear regression curve (concentration vs. effect) [52].

#### 4.6.3. Ferric Reducing Antioxidant Power

The FRAP solution was prepared by mixing 1 mL TPTZ (10 mM/L), 1 mL FeCl_3_ (20 mmM/L) and 10 mL sodium acetate buffer (pH 3.6), which was stored at 37 °C. The 50 μL sample diluted to the appropriate concentration and 250 μL FRAP working solution were mixed in 96-well plate, and the absorbance was measured at 593 nm after 10 min of dark reaction at 37 °C. FRAP standard curve was drawn with FeSO_4_ standard solution concentration as ordinate and absorbance as abscissa. Ascorbic acid (V_C_) and quercetin (BHT) was used as antioxidant standard. The FRAP value was expressed as μg of FeSO_4_ equivalents/mg dry extract (DE) [53].

### 4.7. Determination of Bioactive Compounds

#### 4.7.1. Total Phenolic Content

The Folin–Ciocalteu method was used to determine the total phenolic content in the extract. At the condition of room temperature, add 50 μL of standard and samples diluted to appropriate concentrations to a 96-well microtiter plate. Add 125 μL of Folin–Ciocalteu solution diluted to 0.5 mol/L. Add 100 μL of 7.5% (*w*/*v*) Na_2_CO_3_. The same volume of distilled water was added to the control group. The absorbance was measured at 765 nm after reacting in the dark for 30 min. Under the same conditions, measure the absorbance of gallic acid at 10 μg/mL, 20 μg/mL, 40 μg/mL, 60 μg/mL, 80 μg/mL and 100 μg/mL. Take the concentration of gallic acid as the abscissa, and take the absorbance value as the on the ordinate, draw a standard curve, calculate the total phenolic content. The result is expressed as mg gallic acid equivalent (GAE)/g fresh extract (FE), repeated 3 times [54].

#### 4.7.2. Total Flavonoids Content

At the condition of room temperature, 20 μL NaNO_3_ (3%, *w*/*v*) was mixed with 40 μL appropriately diluted samples in a 96-well microtiter plate. After 6 min, 20 μL Al (NO_3_) _3_ (6%, *w*/*v*) was added to react for 6 min. Then, 140 μL NaOH (4%, *w*/*v*) and 70% methanol (containing 1% formic acid) were added, the reaction was continued for 15 min. The absorbance of the mixture was measured at 510 nm. Draw the standard curve with the absorbance of rutin (100 μg/mL, 200 μg/mL, 400 μg/mL, 600 μg/mL, 800 μg/mL and 1000 μg/mL). Take the rutin concentration as the abscissa, the absorbance is the ordinate, the content of total flavonoids was calculated. Results were expressed as mg rutin equivalents (RE)/g fresh extract (FE), repeated 3 times [54].

### 4.8. Data Processing and Analysis

All experiments were repeated 3 times. Execel statistical test data were used. Data are presented as mean ± standard deviation (SD). SPSS v20.0 statistical software was used to test the significant differences of the experimental data (*p* < 0.05), and Origin Pro v9.0 was used for graphing.

## 5. Conclusions

In this study, the phytochemical composition of the essential oils from *P. delavayi* dark-purple, red and yellow flowers were determined. Essential oil extraction yields varied in the different colors of flower petals. Compositions of essential oils extracted from *P. delavayi* flowers showed differential accumulations corresponding to different colors, with 2-(5-Ethenyl-5-methyloxolan-2-yl)propan-2-ol being the predominant phytochemical in dark-purple flowers and copaene being dominant and unique in the yellow flower. Essential oils from different flower colors showed high free radical scavenging activity and strong antioxidant activity, indicating that they may serve as promising sources of natural antioxidants. To the best of our knowledge, this is the first comparative study of the composition and biological activity of essential oils of peony plants with different flower colors, in order to provide data to support for the development of *P. delavayi* flower products and industry. Future studies should also focus on the phytochemicals and their biological activities of the essential oils of *P. delavayi*.

## Figures and Tables

**Table 1:** Common compounds of essential oil for different colors of *P. delavayi* flowers.

Number	Compounds	Relative Content/%		
		Dark-Purple	Red	Yellow
1	Benzyl alcohol	2.24 a ± 0.97	3.24 a ± 1.00	3.44 a ± 0.86
2	Phenethyl alcohol	1.76 ab ± 0.51	0.80 a ± 0.15	2.06 bc ± 0.92
3	(S,3E,7E)-α,α,4,8-Tetramethyl-3,7-cyclodecadiene-1-methanol	0.42 ab ± 0.13	0.38 ab ± 0.17	2.50 c ± 0.35
4	α-cadinol	1.11 a ± 0.38	0.94 ab ± 0.26	4.30 c ± 0.85
5	Phytol	2.15 a ± 0.58	3.48 ac ± 0.73	2.51 ab ± 0.62
6	2-(5-Ethenyl-5-methyloxolan-2-yl)propan-2-ol	20.88 ab ± 1.64	21.81 ab ± 1.04	4.06 c ± 0.82
7	Bicyclo[3.2.2]nona-6,8-dien-3-one	0.60 a ± 0.17	0.79 a ± 0.08	0.60 a ± 0.13
8	Fitone	4.34 c ± 0.52	2.20 ab ± 0.37	2.02 ab ± 0.28
9	Benzaldehyde	0.15 a ± 0.04	0.17 a ± 0.07	0.10 a ± 0.02
10	Trans-Cinnamaldehyde	4.92 a ± 0.64	4.31 a ± 0.81	0.33 b ± 0.06
11	(E)-linalool oxide (pyranoid)	11.90 a ± 1.45	10.85 ac ± 1.26	0.97 b ± 0.14

Note: Different letters lowercase in the same line indicate significant differences (*p* < 0.05).

**Table 2:** Comparison of total phenolic compounds and flavonoids in the essential oils for different colors of *P. delavayi* flowers.

Petal Color	Total Phenolic Content (mg/g)	Total Flavonoids Content (mg/g)
Dark-purple	28.71 ab ± 0.42	10.8 a ± 0.06
Red	23.68 b ± 0.76	9.43 bc ± 0.21
Yellow	18.89 c ± 0.36	5.45 c ± 0.12

Different letters in the table indicate significant differences (*p* < 0.05).

## Data Availability

Not applicable.

## Supplementary Materials

The following supporting information can be downloaded at: https://www.mdpi.com/article/10.3390/molecules27093000/s1, Table S1: Physical and chemical properties of essential oil from *P. delavayi* flowers with different colors; Table S2: Essential oil composition of *P. delavayi* flower with different colors; Table S3: Eigenvalues and contribution rates of principal components; Table S4: Principal component load matrix; Figure S1: GC-MS total ion chromatogram of essential oil from *P. delavayi* flowers with different colors (A: Dark-purple petals B: Red petals C: Yellow petals); Figure S2: Test materials of dark-purple, red and yellow flowers of *P. delavayi*.

## Sample Availability

Samples of the compounds are available from the authors.

## References

[1] Shi Q.Q., Zhou L., Wang Y. (2015). Transcriptomic Analysis of *Paeonia delavayi* Wild Population Flowers to Identify Differentially Expressed Genes Involved in Purple-Red and Yellow Petal Pigmentation. PLoS ONE.

[2] Hong D., Pan K. (1999). Taxonomical history and revision of Paeonia sect. Moutan (Paeoniaceae). J. Syst. Evol..

[3] Hua M., Ma H., Tan R., Yuan X., Chen J., Yang W. (2018). Determination of anthocyanins and flavonols in *Paeonia delavayi* by high-performance liquid chromatography with diode array and mass spectrometric detection. Anal. Lett..

[4] Wu S.H., Chen Y.W., Li Z.Y., Yang L.Y., Li S.L. (2009). Chemical constituents from the root bark of *Paeonia delavayi*. Chem. Natur. Compd..

[5] Tao A.E., Zhao F.Y., Xia C.L. (2019). The complete chloroplast genome sequence of the medicinal plant *Paeonia delavayi* Franchet. (Paeoniaceae). Mitochondrial DNA Part B-Resour..

[6] Zhang J.M., Liu J., Sun H.L., Yu J., Wang J.X. (2012). Nuclear and chloroplast SSR markers in *Paeonia delavayi* (paeoniaceae) and cross-species amplification in *P. ludlowii*. Am. J. Bot..

[7] Li K., Zheng B.Q., Wang Y., Guo X. (2013). Study on pollination biology of *paeonia delavayi* (peaoniaceae). Acta Hortic..

[8] Tan S.L., Peter M.H., Qin H.T., Ye L.J., Zou J.Y., Gao L.M. (2019). Development of polymorphic microsatellite markers for tree peony *paeonia delavayi* (paeoniaceae) using ddrad-seq data. Mol. Biol. Rep..

[9] Luo X.N., Yuan M., Li B.J., Li C.Y., Zhang Y.L., Shi Q.Q. (2020). Variation of floral volatiles and fragrance reveals the phylogenetic relationship among nine wild tree peony species. Flavour Fragr. J..

[10] Shi Q.Q., Li L., Zhou L., Wang Y. (2018). Morphological and Biochemical Studies of the Yellow and Purple-red Petal Pigmentation in *Paeonia delavayi*. Hortscience.

[11] Wang J., Lewis D., Shi R., McGhie T., Wang L., Arathoon S., Schwinn K., Davies K., Qian X.H., Zhang H.B. (2022). The colour variations of flowers in wild *Paeonia delavayi* plants are determined by four classes of plant pigments. N. Z. J. Crop. Hortic. Sci..

[12] Monahan A.H. (2000). Nonlinear principal component analysis by neural networks: Qheory and application to the lorenz system. J. Clim..

[13] Yuan W.Q., Hu J.Z., Wu Y., Yin L.Q., Han X., Wang X.X., Lv Z.L. (2020). Essential oil composition of *Xanthoceras sorbifolium* flower. J. Beijing For. Univ..

[14] Wu Y., Hu J.Z., Han X., Yin L.Q., Yuan W.Q., Zhao Q.L., Lv Z.L. (2020). Comparison in essential oil components of different varieties at varied altitudes of *Paeonia rockii*. J. Beijing For. Univ..

[15] Zhang X.Y., Wu Y., Han X., Yan Z.X., Yuan W.Q., Lv Z.L. (2020). Characterization of volatile compounds in five blueberry varieties using purge and trap coupled to gas chromatography-mass spectrometry. Ital. J. Food Sci..

[16] Yu J., Chen G.L., Yang L.Q., Wang Y.K., Gao Y.Q., Fu N.L. (2017). Study on Purification and Antioxidant Activity of Polyphenols from the Flower of *Paeonia lactiflora* Palla. Food Res. Dev..

[17] Xue Y.Q., Liu R., Xue J.Q., Wang S.L., Zhang X.X. (2021). Genetic diversity and relatedness analysis of nine wild species of tree peony based on simple sequence repeats markers. Hortic. Plant. J..

[18] Sun J.Y., Zhang X.X., Niu L.X., Zhang Y.L. (2017). Chemical compositions and antioxidant activities of essential oil extracted from the petals of three wild tree peony species and eleven cultivars. Chem. Biodivers.

[19] Li S., Wang C.Z., Tang X.X., Wang X.H., Zhou X. (2015). Study on the Physical-chemical Properties and Components of Peony Essential Oil Extracted by Different Extraction Methods. Food Ind..

[20] Jia P.P., Wang X.C. (2017). Analysis of odor components derived from Chinese soft-shelled turtle (*Trionyx sinensis*) calipash cooked by steaming and boiling. Sci. Technol. Food Ind..

[21] Vella F.M., Calandrelli R., Cautela D., Fiume I., Laratta B. (2020). Chemometric screening of fourteen essential oils for their composition and biological properties. Molecules.

[22] Mischko W., Hirte M., Fuchs M., Mehlmer N., Brück T.B. (2018). Identification of sesquiterpene synthases from the basidiomycota coniophora puteana for the efficient and highly selective β-copaene and cubebol production in E. coli. Microb. Cell Factories.

[23] Xu J., Wang M.L., Li T.T., Rao Z., Zhang Y., Sun W., Wen X., Wang Z., Ding P., Yuan W. (2014). Composition and content of tobacco (*Nicotiana tabacum* L.) leaf cuticular waxes. Acta Agric. Boreali-Occident. Sin..

[24] Chen X.Q., Zhang H.Y., Xu P.Z. (2008). Analysis of content of anthocyanidins, proanthocyanidins and flavone in color rice. Molecular Plant. Breeding..

[25] Dai S.L., Hong Y. (2016). Molecular breeding for flower colors modification on ornamental plants based on the mechanism of anthocyanins biosynthesis and coloration. Sci. Agric. Sin..

[26] Guo X.F., Jia X.G., Ni H., Sun Q.W., Li X.J. (2012). Study on the extraction and purification technology of the active ingredient from *Cistanche tubulosa*. J. Xinjiang Med. Univ..

[27] Chun J.Y., Jo Y.J., Bjrapha P., Choi M.J., Min S.G. (2015). Antimicrobial Effect of alpha- or beta-Cyclodextrin Complexes with Trans-Cinnamaldehyde Against Staphylococcus aureus and Escherichia coli. Dry. Technol..

[28] Bektas T., Munevver S., Askin A., Dimitra D., Moschos P., Atalay S. (2005). Antioxidative activity of the essential oils of *Thymus sipyleus* subsp. *sipyleus* var. *sipyleus* and *Thymus sipyleus* subsp. *sipyleus* var. *rosulans*. J. Food Eng..

[29] Burt S. (2004). Essential oils: Their antibacterial properties and potential applications in foods—a review. Int. J. Food Microbiol..

[30] Prasad S., Gupta S.C., Tyagi A.K. (2017). Reactive oxygen species(ROS)and cancer: Role of antioxidative nutraceuticals. Cancer Lett..

[31] Dröge W. (2002). Free radicals in the physiological control of cell function. Physiol. Rev..

[32] Saad D., Yasser T., Muhammad I., Mohamed A., Mohammed E. (2015). The anticancer, antioxidant and antimicrobial properties of the sesquiterpene β-caryophyllene from the essential oil of *aquilaria crassna*. Molecules.

[33] Gullón B., Gullón P., Lú-Chau T.A. (2017). Optimization of solvent extraction of antioxidants from *Eucalyptus globulus* leaves by response surface methodology: Characterization and assessment of their bioactive properties. Ind. Crops Prod..

[34] Avanço G.B., Ferreira F.D., Bonfim N.S. (2017). *Curcuma longa*, L. essential oil composition, antioxidant effect, and effect on Fusarium verticillioides, and fumonisin production. Food Control..

[35] Delgado-Andrade C., Rufian-Henares J.A., Morales F.J. (2005). Assessing the antioxidant activity of Melanoidins from coffee brews by different antioxidant methods. J. Agric. Food Chem..

[36] Ardestani A., Yazdanparast R. (2007). Antioxidant and free radical scavenging potential of *Achillea santolina* extracts. Food Chem..

[37] Bao Y.T., Qu Y., Li J.H., Li Y.F., Ren X.D., Maffucci K.G., Li R.P., Wang Z.G., Zeng R. (2018). In Vitro and In Vivo Antioxidant Activities of the Flowers and Leaves from *Paeonia rockii* and Identification of Their Antioxidant Constituents by UHPLC-ESI-HRMS via Pre-Column DPPH Reaction. Molecules.

[38] Jin Y.S., Chen M.L., Jin Y.Z., Tao J. (2012). In vitro free radical scavenging activities and active constituents from *Paeonia lactiflora* flowers. J. Yangzhou Univ. (Agric. Life Sci. Ed.).

[39] Li H.L., Gao X., Xu F.L., Chen H.K., Wang W.L. (2017). Chemical composition and antioxidant activities of Essential oil from *Paeonia lactiflora* flowers. J. Northwest. AF Univ. (Nat. Sci. Ed.).

[40] Chen G.L., Chen S.G., Xie Y.Q., Chen F., Zhao Y.Y., Luo C.X., Gao Y.Q. (2015). Total phenolic, fl avonoid and antioxidant activity of 23 edible flowers subjected to in vitro digestion. J. Funct. Foods.

[41] Shang P.P., Jia M.X., Liu A.Q., Jiang X.R., Liu Y. (2016). Reaserch on the Bioactive Compounds and Antioxidant Activity of Petals from Different Cultivars of *Paeonia lactiflora*. Plant. Physiol. J..

[42] Maham I., Bushra A., Faqir M., Ali S., Muhammad F.A., Irfan H., Kashif S., Hosh M. (2021). Antioxidant and Wound Healing Potential of Essential Oil from *Citrus reticulata* Peel and its Chemical Characterization. Curr. Pharm. Biotechnol..

[43] Jang H.D., Chang K.S., Chang T.C., Hsu C.L. (2010). Antioxidant potentials of buntan pumelo (*Citrus grandis* Osbeck) and its ethanolic and acetified fermentation products. Food Chem..

[44] Gürkan S., Gurbet Ç., Erhan G., Aslı S. (2016). Essential oil composition, antioxidant activity and phenolic content of endemic *Teucrium alyssifolium* Staph. (Lamiaceae). Nat. Prod. Res..

[45] Li D.X., Qian J., Huang D.Z., Guo X., Tian M., Zeng D.Q. (2018). Analysis of the Antioxidant and Antifungal Activities of the Essential oil of *Laurocerasus phaeosticta*. Chin. J. Trop. Agric..

[46] Antonella R., Andrea M., Danilo P., Angela A., Benedetta E., Antonella F., Cinzia S., Piras A. (2017). Chemical composition of *Lycium europaeum* fruit oil obtained by supercritical CO_2_ extraction and evaluation of its antioxidant activity, cytotoxicity and cell absorption. Food Chem..

[47] Qiu W., Su W., Cai Z., Dong L., Wu Z. (2020). Combined Analysis of Transcriptome and Metabolome Reveals the Potential Mechanism of Coloration and Fruit Quality in Yellow and Purple *Passiflora edulis* Sims. J. Agric. Food Chem..

[48] Fan J.L., Zhu W.X., Kang H.B., Ma H.L., Tao G.J. (2012). Flavonoid constituents and antioxidant capacity in flowers of different Zhongyuan tree penoy cultivars. J. Funct. Foods.

[49] Yu H., Ma W.P., Liu Y.P., Li J.X., Liu J.M. (2015). Analysis of Volatile Components in Peony Essence Oil by Headspace Gas Chromatography-Mass Spectrometry. Food Sci..

[50] Liu J.Y., Yin G.Y., Zhang H.J., Wu Y.S., Xu S.T., Wei J. (2013). Comparison of essential oils from *Macadamia ternifolia* flowers by supercritical carbon dioxide fluid extraction and simultaneous distillation extraction. J. Yunnan Univ..

[51] Lin L.J., Huang X.B., Liu M.J., Li J.H. (2019). Composition of Essential oils and Hydrosols Acquired from *Alpinia officinarum* Hance by Supercritical CO_2_ Extraction and Steam Extraction. Chin. J. Trop. Crops.

[52] Ghane S.G., Attar U.A., Yadav P.B., Lekhak M.M. (2018). Antioxidant, anti-diabetic, acetylcholinesterase inhibitory potential and estimation of alkaloids (lycorine and galanthamine) from Crinum species: An important source of anticancer and anti-Alzheimer drug. Ind. Crops Prod..

[53] Liao H., Banbury L. (2015). Different Proportions of Huangqi (*Radix Astragali* Mongolici) and Honghua (*Flos Carthami*) Injection on α-Glucosidase and a α-Amylase Activities. Evid. -Based Complementray Altern. Med..

[54] Wang Z.X., Lin Q.Q., Tu Z.C., Zhang L. (2020). The influence of in vitro gastrointestinal digestion on the *Perilla frutescens* leaf extract: Changes in the active compounds and bioactivities. J. Food Biochem..

