# Supplementary material for: Phytochemical Compositions and Antioxidant Activities of Essential Oils Extracted from the Flowers of Paeonia delavayi Using Supercritical Carbon Dioxide Fluid
Source: Molecules. 2022 May 7;27(9):3000. doi: 10.3390/molecules27093000 (PMC9099896; doi:10.3390/molecules27093000)
Supplement: Supplementary file 1 [file molecules-27-03000-s001.zip › molecules-1694722-supplementary.pdf]

Content：

Table S1. Physical and chemical properties of essential oil from *P. delavayi* flowers with different colors
Table S2. Essential oil composition of *P. delavayi* flower with different colors
Table S3. Eigenvalues and contribution rates of principal components
Table S4. Principal component load matrix
Figure S1. GC-MS total ion chromatogram of essential oil from *P. delavayi* flowers with different colors (A: Dark-purple petals B:Red petals C:Yellow petals)
Figure S2. Test materials of dark-purple , red and yellow flowers of *P. delavayi*

Table S1 Physical and chemical properties of essential oil from *P. delavayi* flowers with different colors

Petal Color	Yield/%	Appearance	Tincture	Fragrance	Relative density	Optical rotation	Diopter
Dark-purple	0.78 ± 0.02a	Clear liquid	Milk white	Superfine fragrant	0.90 ± 0.035ab	-3.78° ± 0.33b	1.47 ± 0.14a
Red	0.64 ±0.01ab	Clear liquid	Milk white	Superfine fragrant	0.85 ± 0.028a	-2.98° ± 0.77b	1.46 ± 0.26b
Yellow	0.58 ±0.07a	Clear liquid	Pale Yellow	Delicate fragrance	0.80 ± 0.046bc	-3.14° ± 0.48ab	1.46 ±0.07ab

Notes：mean ± SD (n = 3). Identical letters indicate not significant difference (P > 0.05).

Table S2 Essential oil composition of *P. delavayi* flower with different colors

Compound type	Retention time/min	Compounds	Molecular formmula	Relative content/%		
				Dark-purple	Red	Yellow
Alcohol	3.78	Cyclobutanemethanol	$C_5H_{10}O$	0.00b	0.00b	0.20a±0.02
	4.01	(S,S)-2,3-Butanediol	$C_4H_{10}O_2$	0.00a	0.10b±0.03	0.00a
	5.30	Leaf alcohol	$C_6H_{12}O$	0.00a	0.34b±0.04	0.00a
	5.62	1-Hexanol	$C_6H_{14}O$	0.00a	0.14b±0.01	0.00a
	5.28	Trans-3-Hexen-1-ol	$C_6H_{12}O$	0.35a±0.07	0.00b	0.38ac±0.04
	14.21	Benzyl alcohol	C_7H_8O	2.24a±0.97	3.24a±1.00	3.44a±0.86
	15.71	Dl-1-Phenethylalcohol	$C_8H_{10}O$	0.00b	0.61ab±0.04	5.97c±0.83
	17.26	7-Octen-2-ol, 2-Methyl-6-methylene	$C_{10}H_{18}O$	0.00b	1.70a±0.41	0.00b
	18.02	Linalool	$C_{10}H_{18}O$	0.00a	1.00c±0.26	0.58b
	18.58	Phenethyl alcohol	$C_8H_{10}O$	1.76ab±0.51	0.80a±0.15	2.06bc±0.92
	23.00	2,6-Dimethyl-3,7-octadiene-2,6-diol	$C_{10}H_{18}O_2$	0.00a	0.00a	0.24b±0.08
	24.98	3-Phenyl-1-propanol	$C_9H_{12}O$	2.68a±0.43	2.31ac±0.61	0.00b
	25.83	2-(4-methylidenecyclohexyl)prop-2-en-1-ol	$C_{10}H_{16}O$	0.00a	0.00a	2.54b±0.92
	26.30	Lavandulol	$C_{10}H_{18}O$	0.05ab±0.03	0.00b	0.00b
	26.21	Geraniol	$C_{10}H_{18}O$	0.00a	0.00a	1.84b±0.41
	26.87	4-Isopropyl-1,5-cyclohexadiene-1-methanol	$C_{10}H_{16}O$	0.00a	0.00a	0.53b±0.08
	27.33	(βR,2S,5S)-β,5-Dimethyl-5β-vinyltetrahydrofuran-2α-ethanol	$C_{10}H_{18}O_2$	0.29a±0.03	0.18ab±0.08	0.00c
	27.86	Epoxydihydrolinalool	$C_{10}H_{18}O_3$	1.21a±0.28	0.60b±0.07	0.00b
	29.03	Cinnamyl alcohol	$C_9H_{10}O$	1.36a±0.63	0.29b±0.13	0.00b
	29.15	β-Methylenephenethyl alcohol	$C_9H_{10}O$	0.00a	5.60b±1.05	0.00a
	30.40	2,6-dimethyloct-7-ene-2,6-diol	$C_{10}H_{20}O_2$	0.86a±0.24	1.49bc±0.83	0.00b
	30.98	4-(Allyloxy)-2-methyl-2-pentanol	$C_9H_{18}O_2$	0.00a	0.30b±0.07	0.00ac
	31.13	3-Ethyl-2-methyl-2-heptanol	$C_{10}H_{22}O$	0.00a	0.02b±0.02	0.00ab
	32.62	2,2-Difluoro-1,3-benzodioxole-4-carbonyl chloride	$C_{10}H_{18}O_2$	0.33a±0.09	0.00b	0.00bc
	38.97	(S,3E,7E)-α,α,4,8-Tetramethyl-3,7-cyclodecadiene-1-methanol	$C_{15}H_{26}O$	0.42ab±0.13	0.38ab±0.17	2.50c±0.35
	40.20	1,4-Dichloro-2-butanol	$C_4H_8Cl_2O$	0.00a	0.00a	0.12ab±0.08
	42.52	(1S)-1,6-Dimethyl-4β-isopropyl-1,2,3,4,4aβ,7,8,8aβ-octahydronaphthalene-1β-ol	$C_{15}H_{26}O$	0.00a	0.94b±0.15	0.00a

	42.91	10-epi-γ-eudesmol	$C_{15}H_{26}O$	0.00a	0.18ab±0.07	0.00a
	42.99	α-cadinol	$C_{15}H_{26}O$	1.11a±0.38	0.94ab±0.26	4.30c±0.85
	43.45	2-(4a,8-Dimethyl-2,3,4,5,6,7-hexahydro-1H-naphthalen-2-yl)propan-2-ol	$C_{15}H_{26}O$	0.00a	0.00a	0.48ab±0.13
	43.463	10-epi-.gamma.-Eudesmol	$C_{15}H_{26}O$	0.06a±0.03	0.00a	0.00a
	48.72	Cryptomeridiol	$C_{15}H_{28}O_2$	0.00a	3.11b±0.87	0.00a
	48.93	1-(2-Methoxyphenyl)-2-propanol	$C_{10}H_{14}O_2$	0.15ab±0.07	0.00a	0.00a
	50.09	2-Isopropyl-4a-methyl-8-methylenedecahydro-1,5-naphthalenediol	$C_{15}H_{26}O_2$	0.00a	0.51b±0.13	0.00a
	50.07	3-Isopropyl-6,7-dimethyltricyclo[4.4.0.0(2,8)]decane-9,10-diol	$C_{15}H_{26}O_2$	1.05a±0.41	0.00b	1.38ac±0.72
	51.13	4,4-Dimethyladamantan-2-ol	$C_{12}H_{20}O$	0.65a±0.16	0.59a±0.08	0.00b
	58.34	Phytol	$C_{20}H_{40}O$	2.15a±0.58	3.48ac±0.73	2.51ab±0.62
Ester	3.33	Resorcinol monobenzoate	$C_{13}H_{10}O_3$	0.00a	0.00a	0.11ab±0.08
	19.77	3-Methylbut-2-enoic acid, 3-fluorophenyl ester	$C_8H_{20}O_4Si$	0.14a±0.08	0.10a±0.06	0.00c
	16.23	2-(5-Ethenyl-5-methyloxolan-2-yl)propan-2-ol	$C_{13}H_{22}O_4$	20.88ab± 1.64	21.81ab±1.04	4.06c±0.82
	18.06	Phosphonofluoridic acid, (1-methylethyl)-, octyl ester	$C_{11}H_{24}FO_2P$	0.88a±0.25	0.00b	0.00b
	19.77	3-Methylbut-2-enoic acid, 3-fluorophenyl ester	$C_{11}H_{11}FO_2$	0.02a±0.01	0.00a	0.00a
	22.50	Crotonic acid cis-3-hexen-1-yl ester	$C_{10}H_{16}O_2$	0.00a	0.00a	0.12b0.08
	22.84	Methyl salicylate	$C_8H_8O_3$	0.00a	0.00a	0.19ab±0.05
	23.16	Cyclooctyl isopropylphosphonofluoridate	$C_{11}H_{22}FO_2P$	0.00a	5.16b±0.85	0.00a
	23.22	Isopropylphoshonic acid, fluoroanhydride, 1-methylheptyl ester	$C_{11}H_{24}FO_2P$	3.40a±0.62	0.00b	0.00b
	24.77	Butyric acid, 2-phenyl-, 3-methylbut-2-en-1-yl ester	$C_{15}H_{20}O_2$	0.00a	0.00a	0.19ab±0.04
	25.43	2-Chloroethyl benzoate	$C_9H_9ClO_2$	0.00a	0.32b±0.08	0.00a
	25.77	2,3-Dipropyl-cyclopropanecarboxylic acid, ethyl ester	$C_{12}H_{22}O_2$	2.15a±0.38	0.00b	0.00b
	26.31	Acetic acid, 2,2,2-trifluoro-, 5-methyl-2-(1-methylethenyl)-4-hexen-1-yl ester	$C_{12}H_{17}F_3O_2$	0.00a	0.19ab±0.07	0.07a±0.02
	26.44	Ethyl 2-AMinoiMidazole-5-carboxylate	$C_6H_9N_3O_2$	0.00a	0.62b±0.15	0.00a
	26.45	Methyl chlorodifluoroacetate	$C_3H_3ClF_2O_2$	1.15a±0.31	0.00b	0.00b
	30.45	Cyclobutanecarboxylic acid, 2-tetrahydrofurylmethyl ester	$C_{10}H_{16}O_3$	0.00a	0.00a	0.08a±0.02
	31.57	EAthyl 2.4-dioxo-4-p-tolylbutanoate	$C_{13}H_{14}O_4$	0.00a	0.00a	0.25b±0.09
	31.74	(2Z)-2-Butenoic acid methyl ester	C5H8O2	0.00a	0.09ab±0.03	0.00a
	35.23	Oxalic acid, 2-isopropylphenyl octyl ester	$C_{19}H_{28}O_4$	0.00a	0.00a	0.51b±0.13
	35.56	Ethyl3-phenylacrylate	$C_{11}H_{12}O_2$	0.10a±0.04	0.00b	0.00b
	37.26	4-Bromobutanoic acid, 1-adamantylmethyl ester	$C_{15}H_{23}BrO_2$	0.00a	0.00a	0.40b±0.08
	37.29	L-Proline, N-(2-methoxybenzoyl)-, isohexyl ester	$C_{19}H_{27}NO_4$	0.04a±0.01	0.00b	0.00b
	37.66	Pentanoic acid, 5-hydroxy-, 2,4-bis(1,1-dimethylethyl)phenyl ester	C19H30O3	0.00a	0.28bc±0.09	0.42bc±0.12
	38.68	l-Alanine, N-(2,3,4-trifluorobenzoyl)-, methyl ester	$C_{11}H_{10}F_3NO_3$	0.00a	0.03ab±0.01	0.00a
	44.08	2-Furanethanol, α-methyl-, 2-acetate	$C_9H_{12}O_3$	0.00a	0.30bc±0.07	0.00a
	45.76	Methyl pentyl methylphosphonate	$C_7H_{17}O_3P$	0.00a	0.00a	0.16b±0.05
	45.84	Phosphonic acid,methyl-,bis(1-methylheptyl) ester	$C_{17}H_{37}O_3P$	0.29b±0.08	0.00a	0.00a

	48.92	5-(1-Hydroxyethylidene)-2-methyl-1,3-cyclopentadiene-1-carboxylic acid methyl ester	$C_{10}H_{12}O_3$	0.00a	0.00a	0.05ab±0.01
	51.74	Tributyl citrate	$C_{18}H_{32}O_7$	0.00a	0.00a	0.25b±0.08
	51.66	5-Tridecanol, 5-acetate	$C_{15}H_{30}O_2$	0.43bc±0.11	0.00a	0.00a
	51.67	Prop-2-yn-1-yl 2-methylbutanoate	$C_8H_{12}O_2$	0.13a±0.07	0.00b	0.00b
	53.50	Dibutyl phthalate	$C_{16}H_{22}O_4$	0.31a±0.09	0.00b	0.00b
	54.16	Phthalic acid, cyclobutyl tridecyl ester	$C_{25}H_{38}O_4$	0.00a	0.04ab±0.02	0.00a
	54.59	Palmitic acid ethyl ester	$C_{18}H_{36}O_2$	0.08a±0.02	0.04a±0.01	0.00ab
	54.58	Sulfurous acid, nonyl pentyl ester	$C_{14}H_{30}O_3S$	0.00a	0.00a	1.12b±0.24
	56.61	Myristic acid, phenyl ester	$C_{20}H_{32}O_2$	0.00a	0.32b±0.08	0.00a
	57.66	2-Thiopheneacetic acid, 2-ethylcyclohexyl ester	$C_{14}H_{20}O_2S$	0.00a	0.06ab±0.02	0.00a
	61.61	Succinic acid, 1,1,1-trifluoroprop-2-yl 2,4-dimethylpent-3-yl ester	$C_{14}H_{23}F_3O_4$	0.00a	0.11b±0.03	0.00a
	5.60	1-Cyclopropylpropane	C_6H_{12}	0.15ab0.08	0.00a	0.11ab0.06
	17.62	Hexaldehyde diethyl acetal	$C_{10}H_{22}O_2$	0.00a	0.00a	0.08b±0.02
	20.59	1-Bromo-1,2-dichlorocyclopropane	$C_3H_3BrCl_2$	0.13ab±0.05	0.05a	0.00a
	26.59	1,1,3,3,5-Pentamethylcyclohexane	$C_{11}H_{22}$	0.00a	0.00a	0.23ab±0.08
	35.17	2,6,10-trimethyldodecane	$C_{15}H_{32}$	1.09a±0.25	0.87a±0.18	0.00b
	36.18	(±)-β-Copaene	$C_{15}H_{24}$	0.00a	0.00a	20.34b±1.34
	36.75	1-methyl-4-(1-methylethylidene)-2-(1-methylvinyl)-1-vinylcyclohexane	$C_{15}H_{24}$	0.00a	0.00a	1.82b0.27
	41.19	3,3-Dimethylhexane	C_8H_{18}	0.00a	0.13b±0.04	0.13b±0.07
	44.64	N-heptadecane	$C_{17}H_{36}$	0.00a	0.26b±0.08	0.00a
	46.68	2-Methyl-3-(3-methyl-but-2-enyl)-2-(4-methyl-pent-3-enyl)-oxetane	$C_{15}H_{26}O$	0.00a	0.09ab±0.02	0.00a
	48.00	germacrane	$C_{15}H_{30}$	0.00a	0.25b±0.08	0.00a
	50.45	Tri(n-butyl)difluorophosphorane	$C_{12}H_{27}F_2P$	0.00a	0.00a	0.74b±0.13
	51.54	N-nonadecane	$C_{19}H_{4}0$	0.00a	0.94b±0.16	0.00a
	51.13	4,5,6,7-Tetrahydroindazole-3-spirocyclohexane	$C_{12}H_{18}N_2$	0.00a	0.00a	0.80b±0.13
	54.65	N-hexadecane	$C_{16}H_{34}$	0.00a	1.00b±0.36	4.36c±0.78
	54.89	2,2,3,3,5,6,6-Heptamethylheptane	$C_{14}H_{30}$	1.07a±0.27	0.00b	0.00b
Alkanes	56.14	1,2-Dibromoethane	$C_2H_4Br_2$	0.00a	0.06a±0.02	0.00a
	56.90	8,8-Diheptylpentadecane	$C_{29}H_{60}$	0.55a±0.13	0.00b	0.05b±0.02
	57.34	2-Cyclohexyldodecane	$C_{18}H_{36}$	0.00ab	0.09ab±0.03	0.00ab
	57.88	N-Heneicosane	$C_{21}H_{44}$	0.00a	0.64b±0.17	0.00a
	57.70	1-Isopropyl-2,3-dimethylcyclopentane	$C_{10}H_{20}$	0.00a	0.00a	0.20b±0.06
	57.91	Tetracosane, 3-methyl-	$C_{25}H_{52}$	0.00a	0.00a	0.26b±0.09
	60.15	2-ethylbicyclo(2.2.1)heptane	C_9H_{16}	0.00a	0.20a±0.05	0.00a
	60.41	3-Methylpentane	$C_{26}H_{54}$	0.00a	2.93b±0.83	0.00a
	61.63	6,6-Dimethylundecane	$C_{13}H_{28}$	0.00a	0.00a	0.06a±0.02
	3.15	Acetoin	$C_4H_8O_2$	0.00a	0.03a±0.01	0.00a
	20.39	1-Hepten-3-one	$C_7H_{12}O$	0.00a	0.00a	0.06a±0.03
	21.09	4-Chloro-4'-hydroxybutyrophenone	$C_{10}H_{11}ClO_2$	0.11ab±0.07	0.00b	0.14ab±0.09
	28.61	Bicyclo[3.2.2]nona-6,8-dien-3-one	$C_9H_{10}O$	0.60a±0.17	0.79a±0.08	0.60a±0.13

Ketone	36.46	4-Methoxy-3-hydroxyacetophenone	$C_9H_{10}O_3$	0.26a±0.05	0.12a±0.03	0.00b
	43.95	2,3-Dihydro-8-methyl-4H-1-benzothiopyran-4-one	$C_{10}H_{10}OS$	0.00a	0.18b±0.08	0.00a
	43.92	6-Methoxy-4-chromanone	$C_{10}H_{10}O_3$	0.12ab±0.04	0.00b	0.00b
	47.95	3'-Hydroxyacetophenone	$C_8H_8O_2$	0.00a	0.00a	0.10a±0.02
	49.75	Fitone	$C_{18}H_{36}O$	4.34c±0.52	2.20ab±0.37	2.02ab±0.28
	52.33	Carissone	$C_{15}H_{24}O_2$	0.00a	0.27bc±0.11	0.51bc±0.14
	56.77	2,5-dimethyl-3-hexanone	$C_8H_{16}O$	0.02a±0.01	0.00a	0.00a
Aldehyde	4.10	2-methylpent-4-enal	$C_6H_{10}O$	0.16a±0.05	0.00a	1.85c±0.14
	5.19	Trans-2-hexenal	$C_6H_{10}O$	0.14b±0.09	0.00a	0.79bc±0.17
	9.30	Benzaldehyde	C_7H_6O	0.15a±0.04	0.17a±0.07	0.10a±0.02
	14.42	2-Phenyl-2-butenal	C_8H_8O	0.00a	0.20b±0.04	0.00a
	14.26	Salicylaldehyde	$C_7H_6O_2$	0.00a	0.00a	0.49b±0.12
	18.18	1-Nonanal	$C_9H_{18}O$	0.18ab±0.04	0.24ab±0.08	0.00c
	22.56	6,6-dimethylbicyclo[3.1.1]heptane-2-carbaldehyde	$C_{10}H_{16}O$	0.23a±0.07	0.12a±0.04	0.00c
	26.73	Trans-Cinnamaldehyde	C_9H_8O	4.92a±0.64	4.31a±0.81	0.33b±0.06
	28.72	2-Ethylbenzaldehyde	$C_9H_{10}O$	0.18a±0.05	0.00b	0.00b
	29.06	Phenylpropyl aldehyde	$C_9H_{10}O$	8.46a±1.37	0.30bc±0.08	0.00c
	55.37	Pentadecanal	$C_{15}H_{30}O$	0.00a	0.19ab±0.07	0.00a
Terpenes	19.71	IsoliMonene	$C_{10}H_{18}$	0.00a	0.00a	0.13ab±0.05
	24.35	Myrcene	$C_{10}H_{16}$	0.00a	0.00a	1.07b±0.35
	28.23	(E)-3,7-dimethylocta-1,3,6-triene	$C_{10}H_{16}$	0.00a	0.00a	0.58b±0.16
	28.24	3,6,6-Trimethyl-2-norpinene	$C_{10}H_{16}$	0.00a	0.29a±0.04	0.00a
	28.27	2,5-Dimethyl-3-vinyl-1,4-hexadiene	$C_{10}H_{16}$	0.35a±0.11	0.00a	0.00a
	29.08	Cis-Bicyclo[4.2.0]octa-3,7-diene	C_8H_{10}	0.06a±0.02	0.00a	0.00a
	32.24	Cis-muurola-4(14),5-diene	$C_{15}H_{24}$	0.00a	0.17bc±0.05	0.30b±0.08
	33.47	(-)-isocaryophyllene	$C_{15}H_{24}$	0.00a	0.00a	1.66b±0.34
	34.93	Alpha-caryophyllene	$C_{15}H_{24}$	0.00a	0.00a	1.37b±0.25
	36.14	(1E,6E,8S)-1-Methyl-5-methylene-8-isopropyl-1,6-cyclodecadiene	$C_{15}H_{24}$	1.74ab±0.27	1.35ab±0.47	0.00b
	36.76	Bicyclo[3.1.1]hept-2-ene,3	$C_{10}H_{16}$	0.00a	0.18ab±0.09	0.00a
	37.49	C15 H24, Naphthalene, 1,2,3,4,4a,5,6,8a-octahydro-7-methyl-4-methylene-1-(1-methylethyl)-	$C_{15}H_{24}$	0.00a	0.00a	1.06b±0.38
	37.89	(+)-delta-Cadinene	$C_{15}H_{24}$	0.00a	0.00a	0.98b±0.15
	42.52	Cis-8-Isopropylbicyclo[4.3.0]non-3-ene	$C_{12}H_{20}$	0.00a	0.00a	1.24b±0.32
	43.67	2,5,5-Trimethyl-1-hexene	C_9H_{18}	0.00a	0.11ab±0.02	0.00a
	45.86	(1S,8aα)-Decahydro-3aα-methyl-7-methylene-1-isopropyl-4α,8α-epithioazulene	$C_{15}H_{24}S$	0.00a	0.00a	0.25b±0.06
	50.59	7R,8R-8-Hydroxy-4-isopropylidene-7-methylbicyclo[5.3.1]undec-1-ene	$C_{15}H_{24}O$	0.49a±0.08	0.00b	0.00b
	57.75	Z-12-Pentacosene	$C_{25}H_{50}$	0.00a	0.00a	1.93±0.41
Phenols	30.96	(Z)-2-methoxy-4-(prop-1-enyl)phenol	$C_{10}H_{12}O_2$	0.32a±0.08	0.00b	0.00b

	42.484	(1S)-1,2,3,4,4aβ,7,8,8aα-Octahydro-1,6-dimethyl-4β-isopropyl-1-naphthol	$C_{15}H_{26}O$	0.68a±0.13	0.00b	0.00b
Others	3.93	N,N-Dimethylformamide	C_3H_7NO	0.00a	0.49b±0.09	0.00a
	17.33	Pyrrole-2-carboxylic acid	$C_5H_5NO_2$	3.81a±0.54	0.00b	0.00b
	21.75	(E)-linalool oxide (pyranoid)	$C_{10}H_{18}O_2$	11.90a±1.45	10.85ac±1.26	0.97b±0.14
	25.75	4-Hexenoic acid, 2-acetyl-2-methyl-, ethyl ester, (E)- (9CI)	$C_{11}H_{18}O_3$	0.00a	0.89b±0.16	0.00a
	25.90	3,5-Methanocyclopentapyrazole, 3,3a,4,5,6,6a-hexahydro-3a,4,4-trimethyl-	$C_{10}H_{16}N_2$	0.00a	0.56b±0.12	0.00a
	26.44	1,2-Benzenediol, O-(5-chlorovaleryl)-O'-(1-naphthoyl)-	$C_{22}H_{19}ClO_4$	1.01a±0.27	0.00b	0.00b
	28.70	2-phenylpropylamine	$C_9H_{13}N$	0.28a±0.04	0.00b	0.00b
	28.73	Benzenepropanoic acid, 2-methyl-3-nitrophenyl ester	$C_{16}H_{15}NO_4$	0.04a±0.01	0.00b	0.00b
	28.78	1-Indano	$C_9H_{10}O$	0.50a±0.12	0.00b	0.00b
	28.79	Butylbenzene	C10H14	0.00a	3.97b±0.57	0.00a
	28.80	2-Methyl-3-nitrophenyl .beta.-phenylpropionate	$C_{16}H_{15}NO_4$	0.18a±0.05	0.00b	0.00b
	29.79	1,3-Oxathiolane, 2,2-dimethyl-	C5H10OS	0.00a	0.04a±0.01	0.00a
	30.63	2,7-Octadiene-1,6-diol, 2,6-dimethyl-, (2Z)-	$C_{10}H_{18}O_2$	0.19a±0.03	0.00b	0.19a±0.05
	31.51	2,7-Octadiene-1,6-diol,2,6-dimethyl-	$C_{10}H_{18}O_2$	1.54a±0.27	2.06b±0.45	0.00c
	33.48	Bicyclo[5.2.0]nonane, 4-ethenyl-4,8,8-trimethyl-2-methylene-	$C_{15}H_{24}$	0.22ab±0.05	0.23ab±0.03	0.00c
	34.94	Cyclopenta[c]pyrrole-1,3(2H,4H)-dione, 5,6-dihydro-4-hydroxy-2-methyl-	$C_8H_9NO_3$	0.80a±0.14	0.00b	0.00b
	35.09	Acetamide, N-(5-methoxy-2-pyrimidinyl)-	$C_7H_9N_3O_2$	0.00a	0.36b±0.09	0.00a
	35.45	Decane, 3,8-dimethyl-	$C_{12}H_{26}$	0.00a	0.00a	0.29b±0.07
	36.75	Cyclohexane, 1-ethenyl-1-methyl-2-(1-methylethenyl)-4-(1-methylethylidene)-	$C_{15}H_{24}$	0.26a±0.04	0.00b	0.00b
	37.08	Cyclobutane, 1,2-di-1-propen-1-yl-	$C_{10}H_{16}$	0.00a	0.00a	0.04a0.01
	37.81	11-Oxatetracyclo[4.2.1.1(2,5).1(3,9)]undec-7-en-10-one, 4-bromo-	$C_{10}H_9BrO_2$	1.08a±0.21	0.00b	0.00b
	37.89	2,4,5-Imidazolidinetrione, 1-methyl-3-phenyl-	$C_{10}H_8N_2O_3$	0.00a	0.10b±0.04	0.00a
	38.63	Benzenecarboimidic acid, N-(3,4-dichlorobenzyloxy)-	$C_{14}H_{11}Cl_2NO_2$	0.00a	0.00a	0.08a±0.02
	42.08	2,5-Cyclohexadien-1-one, 2,4,4-trimethyl-3-(3-oxo-1-butenyl)-, (E)- (9CI)	$C_{13}H_{16}O_2$	0.00a	0.12b±0.04	0.00a
	42.68	Naphthalene, 1,2,3,4,4a,5,6,7-octahydro-4-methyl-7-methylene-1-(1-methylethyl)-, (1S,4R,4aS)-	$C_{15}H_{24}$	0.00a	0.00a	0.32b±0.09
	44.03	5-Amino-1H-imidazol-4-carbonitrile	$C_4H_4N_4$	0.19a±0.07	0.00b	0.00b
	44.12	.Alpha.-(Trifluoromethyl)benzyl alcohol,1-methylpropyl ether	$C_{12}H_{15}F_3O$	0.00a	0.00a	0.10a±0.03
	45.74	Benzamide, 2-hydroxy-N-2-thiazolyl-	$C_{10}H_8N_2O_2S$	0.00a	0.02a±0.01	0.00a
	45.14	Isocapronitrile	$C_6H_{11}N$	0.03a±0.01	0.00a	0.00a
	45.86	Ethanone, 1-[2-[3-methyl-3-(5-methyl-2-furanyl)butyl]-2-oxiranyl]-	$C_{14}H_{20}O_3$	0.00a	0.07a±0.02	0.00a
	46.63	1,3-Cyclohexanediol, 4,6-dimethyl-2-nitro-, monoacetate (ester), [1S-(1α,2β,3α,4β,6β)]- (9CI)	$C_{10}H_{17}NO_5$	0.08a±0.03	0.00a	0.00a
	47.23	2-Naphthalenemethanol, decahydro-8-hydroxy-α,α,4a,8-tetramethyl-	$C_{15}H_{28}O_2$	2.81a±0.54	0.00b	0.00b

47.25	2-Naphthalenemethanol, decahydro-8-hydroxy-α,α,4a,8-tetramethyl-	$C_{15}H_{28}O_2$	0.00a	0.83ab±0.17	7.90c±1.24
47.91	2,4-Pyrimidinediamine, 6-methyl- (9CI)	$C_5H_8N_4$	0.04a±0.01	0.00a	0.00a
48.97	Pyrazine, 2,5-dimethyl-3-(2-propenyl)-	$C_9H_{12}N_2$	0.00a	0.08a±0.03	0.00a
49.48	1-Vinyl-1,2,4-triazole	$C_4H_5N_3$	0.01a±0.01	0.00a	0.00a
50.60	Benzenepropanoic acid, α,5-dimethyl-2-(1-methylethyl)-	$C_{14}H_{20}O_2$	0.00a	0.00a	0.32b0.08
50.62	Bicyclo[5.3.1]undec-1-en-8-ol, 7-methyl-4-(1-methylethylidene)-, [7S-(7R*,8R*)]- (9CI)	$C_{15}H_{24}O$	0.00a	0.42b±0.12	0.00a
50.91	2-Nitrothiophene	$C_4H_3NO_2S$	0.03a±0.01	0.00a	0.00a
52.32	2H-1-Benzopyran-2-one, 6-acetyl-7-(acetyloxy)-4-methyl-	$C_{14}H_{12}O_5$	0.16ab±0.07	0.00b	0.00b
53.51	1(3H)-Isobenzofuranone, 3-ethyl-7-hydroxy-, (3S)-	$C_{10}H_{10}O_3$	0.00a	0.00a	0.38b±0.08
53.54	1,3-Cyclopentadiene, 5,5-dimethyl-1,2-Dipropyl-	$C_{13}H_{22}$	0.00a	0.44b±0.15	0.00a
54.91	Benzene, 1-acetyl-3-ethyl-2-(2-ethenyl-6-ethylphenylazo)-	$C_{20}H_{22}N_2O$	0.00a	0.14b±0.07	0.00a
55.44	1-Benzothiophene-3-carboxylic acid	$C_9H_6O_2S$	0.05a±0.02	0.00a	0.08a±0.03
56.97	2-Propen-1-ol, 3-phenyl-, benzoate, (2Z)- (9CI)	$C_{16}H_{14}O_2$	0.11ab±0.09	0.00a	0.00a
57.63	1H-Imidazole, 4,5-dihydro-2-(1-methylethyl)-	$C_6H_{12}N_2$	0.55a±0.14	0.00b	0.00b
57.80	Dimethyltrifluorophosphorane	$C_2H_6F_3P$	0.27a±0.09	0.00b	0.00b
58.64	Dodecanamide	$C_{12}H_{25}NO$	0.60a±0.18	0.00b	0.00b
58.81	Oleamide	$C_{18}H_{35}NO$	0.00a	0.00a	2.28b±0.51
59.36	2(5H)-Furanone, 5-(1-methylethyl)-	$C_7H_{10}O_2$	0.06a±0.01	0.12ab±0.03	0.00a
60.31	1,4-Dioxaspiro[4.6]undecane, 6-chloro-	$C_9H_{15}ClO_2$	0.00a	0.00a	0.35b±0.04
61.60	Octane, 2,6,6-trimethyl-	$C_{11}H_{24}$	0.00a	0.00a	0.04a±0.01

Different letters represent significant differences between samples ($p \leqslant 0.05$)

Table S3 Eigenvalues and contribution rates of principal components

Principal factor	Eigenvalue	Variance contribution rate /%	Cumulative contribution rate/%
1	7.428	67.528	67.528
2	3.572	32.472	100.00

Table S4 Principal component load matrix

Number	Compounds	Component	
		1	2
1	Benzyl alcohol	-0.546	0.838
2	Phenethyl alcohol	-0.755	-0.655
3	(S,3E,7E)-α,α,4,8-Tetramethyl-3,7-cyclodecadiene-1-methanol	-0.996	0.086
4	α-cadinol	-0.998	0.058
5	Phytol	0.354	0.935
6	2-(5-Ethenyl-5-methyloxolan-2-yl)propan-2-ol	0.998	-0.056
7	Bicyclo[3.2.2]nona-6,8-dien-3-one	0.586	0.810
8	Fitone	0.471	-0.882
9	Benzaldehyde	0.984	0.177
10	Trans-Cinnamaldehyde	0.975	-0.223

11	(E)-linalool oxide (pyranoid)	0.982	-0.189

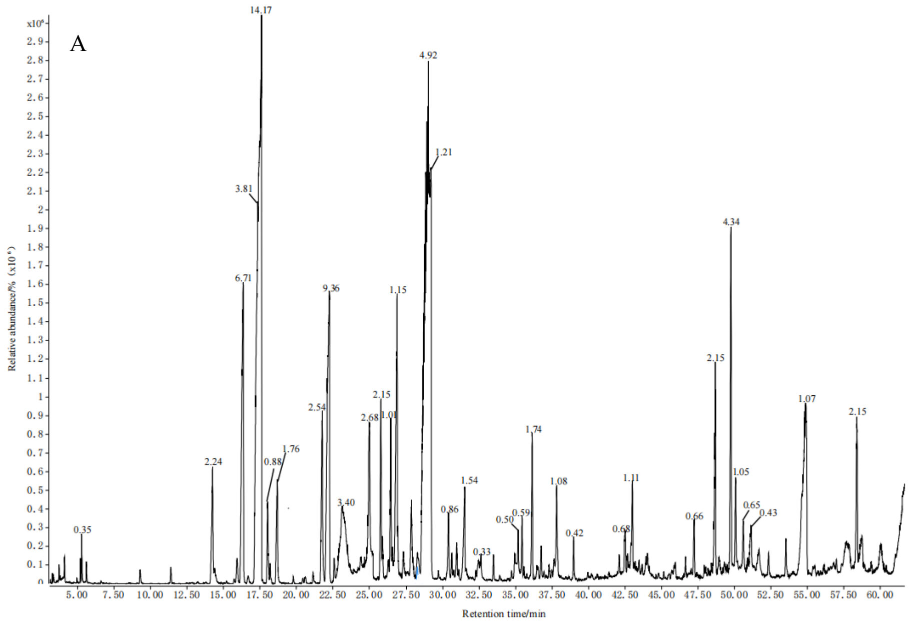

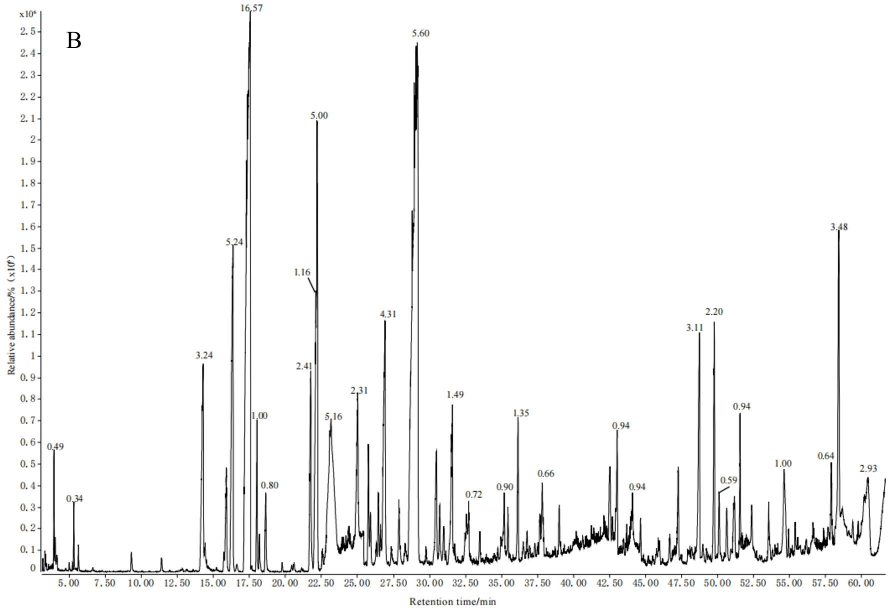

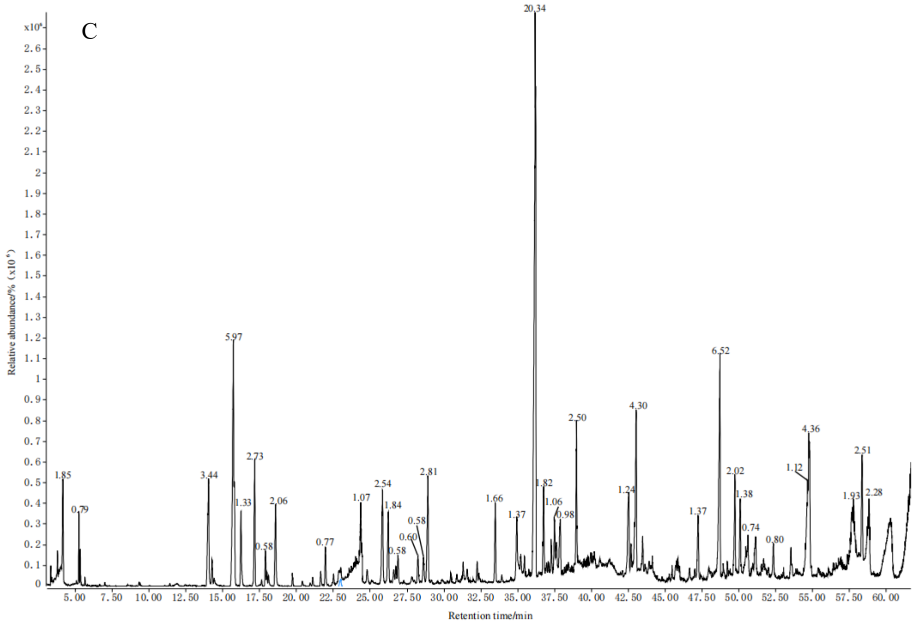

A: Dark-purple petals   B:Red petals   C:Yellow petals

Figure S1 GC-MS total ion chromatogram of essential oil from *P. delavayi* flowers with different colors

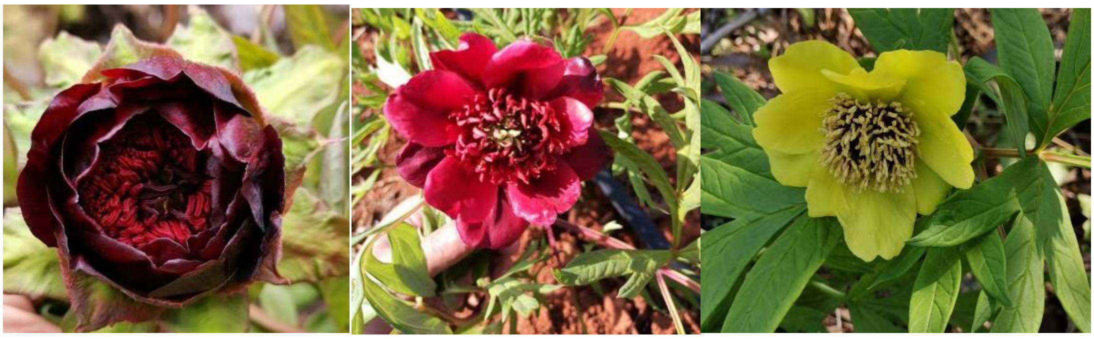

Figure S2 Test materials of dark-purple , red and yellow flowers of *P. delavayi*
